# Correction: Controllable synthesis and enhanced gas sensing properties of a single-crystalline WO_3_–rGO porous nanocomposite

**DOI:** 10.1039/c9ra90069a

**Published:** 2019-10-09

**Authors:** Hao Qin, Tie Liu, Jingyuan Liu, Qi Liu, Xiaoyan Jing, Hongquan Zhang, Guoqing Huang, Jun Wang

**Affiliations:** Key Laboratory of Superlight Material and Surface Technology, Ministry of Education, Harbin Engineering University 150001 PR China zhqw1888@sohu.com +86 451 8253 3026 +86 451 8253 3026; School of Automation, Harbin Engineering University 150001 PR China; Handan Purification Equipment Research Institute Handan 056027 P. R. China

## Abstract

Correction for ‘Controllable synthesis and enhanced gas sensing properties of a single-crystalline WO_3_–rGO porous nanocomposite’ by Qin Hao *et al.*, *RSC Adv.*, 2017, **7**, 14192–14199.

The authors regret that the name of one of the authors (Hao Qin) was shown incorrectly in the original article. The corrected author list is as shown above.

The Royal Society of Chemistry apologises for these errors and any consequent inconvenience to authors and readers.

## Supplementary Material

